# STI/HIV risk prediction model development—A novel use of public data to forecast STIs/HIV risk for men who have sex with men

**DOI:** 10.3389/fpubh.2024.1511689

**Published:** 2025-01-03

**Authors:** Xiaopeng Ji, Zhaohui Tang, Sonya R. Osborne, Thi Phuoc Van Nguyen, Amy B. Mullens, Judith A. Dean, Yan Li

**Affiliations:** ^1^School of Mathematics, Physics and Computing, Centre for Health Research, University of Southern Queensland, Toowoomba, QLD, Australia; ^2^School of Nursing and Midwifery, Centre for Health Research, Institute for Resilient Regions, University of Southern Queensland, Ipswich, QLD, Australia; ^3^School of Psychology and Wellbeing, Centre for Health Research, Institute for Resilient Regions, University of Southern Queensland, Ipswich, QLD, Australia; ^4^School of Public Health, Faculty of Medicine, The University of Queensland, Herston, QLD, Australia

**Keywords:** human immunodeficiency virus, sexually transmissible infections, artificial intelligence, machine learning, risk prediction

## Abstract

A novel automatic framework is proposed for global sexually transmissible infections (STIs) and HIV risk prediction. Four machine learning methods, namely, Gradient Boosting Machine (GBM), Random Forest (RF), XG Boost, and Ensemble learning GBM-RF-XG Boost are applied and evaluated on the Demographic and Health Surveys Program (DHSP), with thirteen features ultimately selected as the most predictive features. Classification and generalization experiments are conducted to test the accuracy, F1-score, precision, and area under the curve (AUC) performance of these four algorithms. Two imbalanced data solutions are also applied to reduce bias for classification performance improvement. The experimental results of these models demonstrate that the Random Forest algorithm yields the best results on HIV prediction, whereby the highest accuracy, and AUC are 0.99 and 0.99, respectively. The performance of the STI prediction achieves the best when the Synthetic Minority Oversampling Technique (SMOTE) is applied (Accuracy = 0.99, AUC = 0.99), which outperforms the state-of-the-art baselines. Two possible factors that may affect the classification and generalization performance are further analyzed. This automatic classification model helps to improve convenience and reduce the cost of HIV testing.

## 1 Introduction

The ongoing challenge of human immunodeficiency virus (HIV) and sexually transmissible infections (STIs) continue to pose substantial global public health concerns. According to the World Health Organization (WHO), there are approximately 374 million new STI cases annually worldwide, while between 1.1 and 2 million individuals were estimated to have been infected with HIV in 2021 (1). STIs/HIV represents a significant burden on the healthcare system of every nation, supporting the need for innovative strategies to be readily implemented if the global target of ending STIs and the HIV epidemic is to be achieved by 2030 (2).

STIs/HIV risk prediction can significantly enhance behavior change and testing rates, which are both critically important for prevention and clinical service delivery. Economical and effective automatic STIs/HIV forecasting has been touted as a potential means for providing urgently needed solutions to improve clinical service delivery and health service optimization (3–8). Machine learning (ML) has been widely applied to healthcare settings and systems, including an increasing application in STIs/HIV risk prediction. Compared with conventional multivariable logistic regression model-based approaches, ML methods can significantly boost the classification prediction of a person at risk of acquiring HIV (9, 10). ML algorithms can also help to determine optimal participants for pre-exposure prophylaxis (PrEP) (11), a form of biomedical prevention, to reduce the likelihood of acquiring HIV if exposed to behaviors that place people at increased HIV risk (e.g., condomless anal intercourse, sharing injecting drug equipment).

Conventionally, HIV infection risks can be assessed through statistical tools. The Cox regression model was introduced by Handan Wand et al. (12) to predict the HIV risk on a dataset collected in KwaZulu Natal, South Africa, over a ten-year period (2002–2012). Seven factors were identified as significant features of determining an HIV risk score. The internal and external experiments based on this model demonstrated its generalizability and robustness. The prediction model produced an area under the curve (AUC) of 79%. This result suggested a screening strategy for HIV prediction/prevention trials by models built from a simple list of questionnaires. However, an inevitable drawback of this statistical model is that its performance is neither adequate nor appropriate for practical clinical applications. Machine-learning-based methods are reliable and effective tools to explore the HIV prediction challenges.

Krakower et al. (13) developed 42 candidate prediction models using machine learning and logistic regression models for the identification of people who would benefit from PrEP. To build an effective model for an incident HIV infection prediction, experiments were conducted on 180 key features from a dataset, which were collected over eight years (2007–2015) by Atrius Health Center (13). Among all tested models, the Least Absolute Shrinkage and Selection Operator (LASSO) algorithm achieved the highest performance with an AUC of 86%, which also indicated the fact that machine learning models helped detect the HIV infection risk for an individual effectively. Bao et al. (10) also compared the effectiveness of machine learning methods and multivariable logistic regression models in predicting STIs/HIV risks among men who have sex with men (MSM), a priority, high-risk group, within Australia from 2011 to 2017. The best AUC values for STIs and HIVs were achieved at 85.8 and 76.3%, respectively, when the GBM model was applied. Xu, X., et al. established a series of machine learning algorithms and statistical analyses to predict STIs/HIV infection over a subsequent 12-month period for both males and females (14). Based on the data provided by the public sexual health center in Melbourne, Australia, from March 2, 2015, to December 31, 2019, individuals who re-tested in the clinic for STIs/HIV were considered, while people who identified as transgender/non-binary and MSM were excluded. Their machine learning-based prediction tool achieved acceptable results in predicting the HIV risk for the next 12 months, with an AUC of 72 and 75% for STIs.

Although many recent studies (4, 15–17) have applied a ML model to predict the risks of having an STI/HIV, only a few studies (18, 19) have paid specific attention to feature extraction techniques for selecting a compelling predictor set (i.e., choosing relevant variables and removing the noise data) to estimate the risks of STIs/HIV. Moreover, most studies only focused on the AUC performance, while other important metrics that lead to a noncomprehensive evaluation (e.g., accuracy, precision) are ignored. Noncomprehensive evaluation might miss critical weaknesses in a model, such as poor performance on certain types of data or in specific contexts. This can lead to overestimating the model’s capabilities. In addition, ML models in previous studies are often trained and tested on the same datasets that were collected from one single clinic/country/region, which would cause problems in usability. Despite unequivocal results from several studies that used AI techniques to determine the risks of STIs/HIV accurately (10, 14, 20), these results are limited in their feasible applications to individuals from other different demographic or ethnic groups, for instance, different countries, and/or from priority/high-risk groups. To resolve the above-identified challenges and critical gaps, in this paper, a novel and high-performance framework was proposed to better select effective features for STIs/HIV predictions, and comprehensive evaluation experiments were carried out to test its classification performance and generalization.

The main contributions of this paper are summarized below:

- A classification framework is proposed for the STIs/HIV risk prediction. Compared with the conventional approaches and the state-of-the-art performance, the proposed framework achieves the best results, when Random Forest algorithm is selected as the classifier. We report the outcomes of this study according to the guidelines of the transparent reporting of a multivariable prediction model for individual prognosis or diagnosis (TRIPOD) reporting (21). The reporting guideline was adapted for AI models as recommended by Collins and Moon (22).
- Analyzing tools to select effective features for STIs/HIV risk prediction are designed for an enhanced culturally (from different ethnic backgrounds) unbiased model.
- More evaluation metrics, including precision, F1-score, etc. are tested in this study to comprehensively evaluate the performance of the proposed framework.
- Classification experiments were conducted based on the subset of the Demographic and Health Surveys Program (DHSP).^1^ Participants being involved in the experiments came from eight countries. Generalization of the proposed methodology was also tested on datasets specific to these eight separated countries. Two factors which can affect the performance are further analyzed.
- The synthetic minority oversampling technique (SMOTE) and a combination of over-sampling the minor class and under-sampling the primary class methods were applied to further improve the classification performance. The best accuracy and AUC of the proposed model are 0.99 and 0.99, respectively, which outperform the existing state-of-the-art. This result demonstrates the probability of self-testing with high reliability, which also may reduce the testing cost.

## 2 Materials and methods

**Figure 1:**
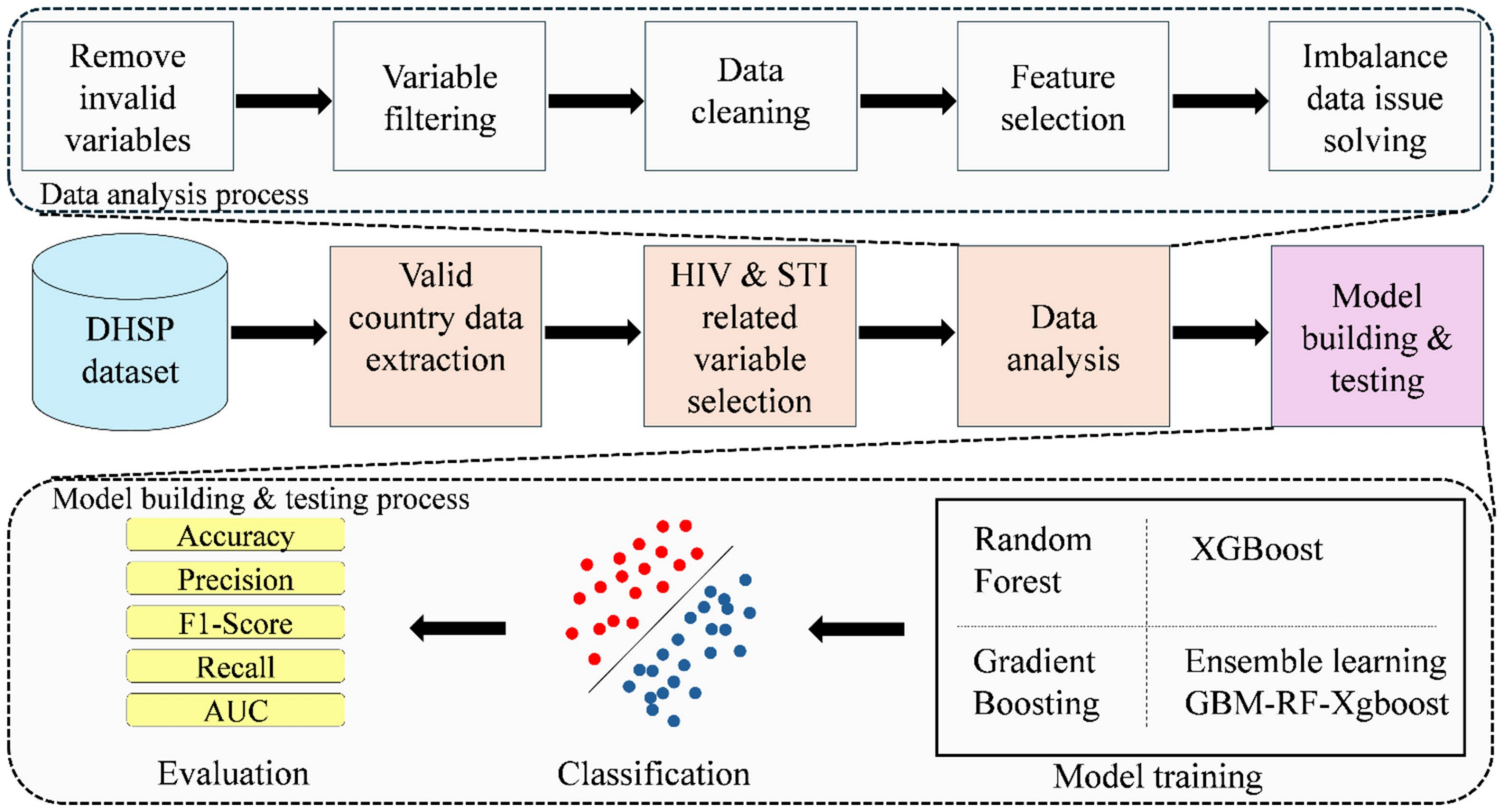
The diagram of the proposed classification framework.

Figure 1 shows the diagram of the proposed method, which consists of four processes in total, namely, extracting valid country data from the whole Demographic and Health Surveys Program (DHSP) dataset, selecting HIV and STI-related variables, data analysis, and model building and testing.

### 2.1 Source of data for the current study

The DHSP (see Footnote 1) is pioneered by the U.S. Agency for International Development (USAID), aiming to enhance policy-making, planning, and management by making these data available on requests for research and/or secondary analysis. This program publishes population, health, HIV, STIs, and nutrition data from approximately 90 countries from six regions (Africa, Asia, Europe, Oceania, Latin America, and the Caribbean). Following the necessary DHS approvals, our project aims to use the DHS data to build an AI framework to predict the infection risks of STIs/HIV among adult men.

### 2.2 Participants

Due to the fact that the DHSP dataset is collected from different regions in different years, the standards followed are different as well. Considering the consistency of data collection standards and STIs/HIV data availability, only countries marked with the HIV data availability are considered initially. Only 37 countries remain after this step, which are further filtered by the feature availability. Even though all 37 countries are related to HIV disease, too many key features for training and prediction are still missing in the collection process for some countries. As a result, participants from eight countries are finally selected for model building and testing, including 9,717 records from the Dominican (2013), 2,028 records from the Dominican Republic (2013), 107,297 records from India (2015), 9,572 records from Haiti (2016), 9,202 records from Haiti (2012), 3,831 records from Guinea (2018), 3,688 records from Guinea (2012), 11,327 records from Ethiopia (2016), 6,648 records from Cameroon (2018), and 5,150 records from Angola (2015). After conducting an extensive literature review (20, 23) and in consultation with experts in public and sexual health, behavior and clinical testing for HIV and STIs and demographic data for men are extracted. A list of detailed variables related to STIs/HIV risk is presented in Table 1.

**Table 1:** Data characteristics considered essential for predicting STI/HIV.

No	Variables	Description	No	Variables	Description
1	mv012	Age	13	mv750	Whether the respondent has ever heard of STIs
2	mv106	Education level	14	mv751	Whether the respondent has ever heard of HIV/AIDS.
3	mv149	Educational attainment	15	mv754cp	Always use condoms during sex
4	mv190	Wealth index	16	mv754dp	Has one sexual partner
5	mv025	Regionality	17	mv766b	Number of lifetime sexual partners
6	mv761	Condom use during last sex with most recent partner	18	mv785	I have heard about other STIs
7	mv501	Current relationship status	19	mv770	I have sought advice or treatment for STI infection
8	mv525	Age at first sexual intercourse	20	mv528	Time since the last sexual intercourse recently (in days)
9	mv536	Most recent sexual activity	21	mv791	Ever provided gifts or other goods in exchange for sex
10	mv714	Current employment	22	mv793	Paid for sex in the last 12 months
11	mv731	Work in last 12 months	23	mv822	(In married heterosexual relationship) The wife feels justified in asking for condom use if the husband has an STI
12	mv717	Occupation group	-	-	-

### 2.3 Data analysis

The data quality has significant effects on prediction performance directly (24). Therefore, the following five steps are applied to help obtain better-quality data.

Step 1: Remove invalid variables: In the Dominican Republic (2013) and Dominica (2013) data sets, three variables, “Current working” (No10-mv714), “Occupation group” (No12 mv717) and ‘Work in last 12 months’ (No14 mv731), are not available. Therefore, those variables are excluded from our input variables for the prediction model.

Step 2: Variable filtering: Some variables are removed since there are not enough samples for model determinations. For example, 94.74% of the data from the question “Sought advice or treatment for STI infection” (No19-mv770) is ‘Nan (Not available or missing data)’. Consequently, this variable is removed from the variable list. Considering variables “Ever heard of STIs” (No13-mv750) and “Whether the respondent has ever heard of AIDS” (No14-mv751), 91% of participants responded “Yes.” However, these variables contribute little to a prediction model since most of the samples have the same value. Thus, we also remove these two variables from our variable list.

Step 3: The data cleaning step removes the rows of data that consisted of Nan (Not available or missing data) values. Each row of these data corresponds with data from a person/subject. Missing data can occur due to the reluctance of people to answer some questions in their surveys, or simply not answering some of the survey questions. Since the reason behind the missing data is unknown, the data for each country is again checked, and rows that consist of Nan values are removed. The original samples are 168,459, which are filtered to 98,449 in this step.

**Figure 2:**
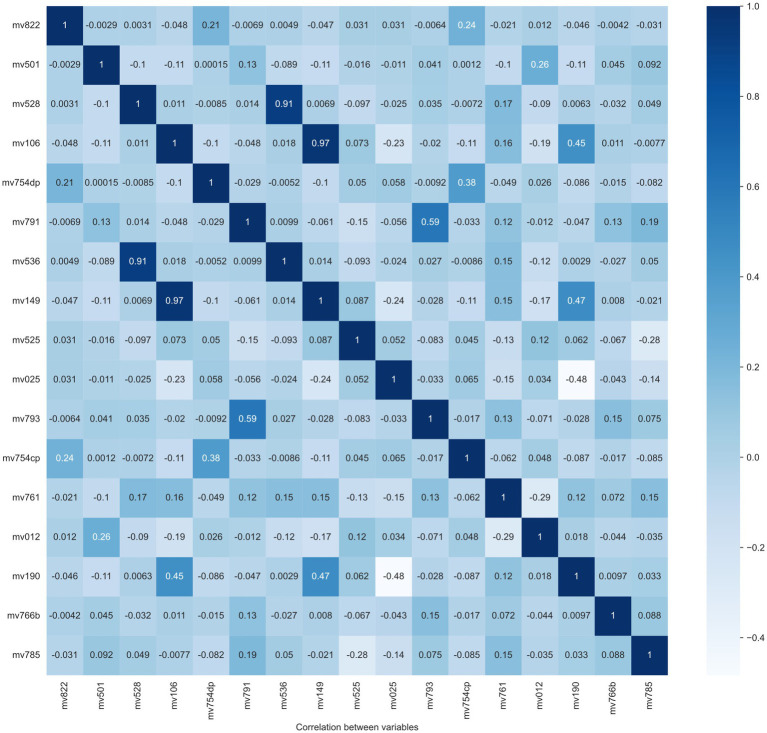
Heatmap of the correlation between all variables.

Step 4: Feature selection. Model performance is normally decided by several factors, like feature quality, number of samples, the algorithm itself, etc. A larger number of features does not always contribute to classifier performance improvement, but it definitely leads to an increase in computational resources. As a result, selecting effective features is a crucial step for model training and resource decrease. In this step, two feature selection methods are applied to exclude the “overlap” or “unimportant” variables, which means linear correlation among features and little contribution to the classification performance, respectively. The overlapped features/variables are determined by calculating the Pearson correlation between variables. From Pearson correlation values, pairs of high correlation features could be found to drop. The Pearson correlation matrix between variables is reported in Figure 2. From this figure, two pairs/couples of high direct correlations (i.e., the similarity is larger than 90%) are found. The first variable pair is “Recent sexual activity” (No9-mv536) and “Time since last intercourse in days” (No21-mv528), and the second variable pair is: “Educational attainment” (No 3-mv149) and “Education level” (No 2-mv106). Due to the high similarity between the two variables in each pair, one variable is removed from each pair. Considering the lower feature importance of the variable No 2 (mv106) and No 9 (mv536), they are removed from our feature list as well.

**Figure 3:**
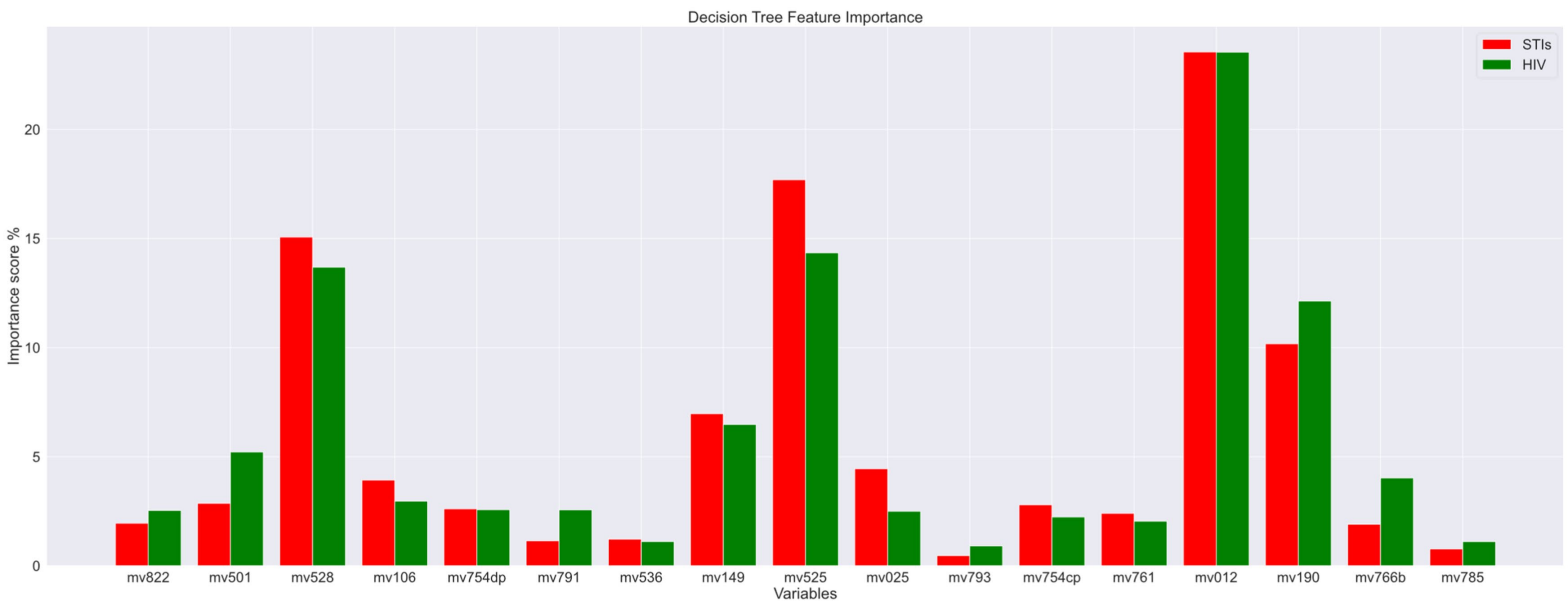
Important feature scores.

The features (variables/features) importance assigns scores to input features based on their significance in predicting the output and variables with low importance scores are removed in this process. These impurity-based feature importance scores, or Gini importance of a Random Forest (RF) algorithm, to be more specific, are obtained by the total (normalized) reduction in the criterion they contribute. As a result, two variables, mv793 (“Paid for sex in the last 12 months”) and mv785 (“Heard about other STIs”), are excluded as their total importance score was minimal. The importance scores are displayed in Figure 3. The red color legend represents the STI risk prediction, and the green color legend signifies the HIV risk prediction. The variable code (mean features) is listed on the x-axis. From this figure, it is evident that “Age” (variable code: mv012) and “Age at first sexual intercourse” (variable code: mv525) emerge as the most critical factors contributing to the risk of both HIV and STIs.

After analyzing data to identify important variables to predict STIs/HIV risks, ten variables are removed, and 13 remaining features to build the model. The final 13 features presented are *age* (mv012), *educational attainment* (mv149), *wealth index* (mv190), *regionality* (mv025), *condom use during last sex with a most recent partner* (mv761), *current marital status* (mv501), *age at first sexual intercourse* (mv525), *Time since last intercourse in days* (mv528), *always use condoms during sex* (mv754cp), *having one sexual partner* (mv754dp), *number of lifetime sexual partner* (mv766b), *giving gifts or other goods in exchange for sex* (mv793), and *the wife is justified in asking for condom use if the husband has an STI* (mv822).

Step 5: Imbalance data issue solving: Due to the fact that samples with STIs/HIV account for a tiny percentage in the dataset (less than 3%) this leads to a data imbalance problem, which can present some difficult challenges in training the machine learning model. Therefore, two preprocessing solutions are applied to solve the imbalanced data problem, including the synthetic minority oversampling technique (SMOTE) and a combination of over-sampling the minor class and under-sampling the primary class (25).

### 2.4 Model building

Random forest (RF) is a classic machine-learning algorithm, which aims to solve both classification problems and regression problems (26). Random forests are constructed by randomly generating several decision trees (DT), and the output of a random forest is determined by voting outputs from all decision trees. Random forests have been widely used in the HIV prediction task (24, 27). Therefore, a standard random forest model is selected for STIs/HIV prediction in this study.

A Gradient Boosting Machine (GBM) model is an ensemble model, whose weak learners will be trained serially, and each weak learner aims to decrease the cumulative model loss from the training process of the previous weak learner (28). Due to its high performance in HIV identification, a GBM model is tested in this study.

Extreme Gradient Boosting (XGBoost) is developed from the boosting algorithm with higher effectiveness and more flexibility (29). Therefore, it has more advantages than other methods to be deployed in edge devices, like smartphones. As a result, it is necessary to evaluate its performance for future employment.

An ensemble model consisting of a standard RF, a GBM, and an XGBoost is designed to develop a classifier, which is expected to outperform all three base learners.

All mentioned models are developed using the built-in machine learning algorithms of the scikit-learn library^2^ through Python programming language.

### 2.5 Experiments setting

For a comprehensive evaluation of all mentioned models, two types of classification experiments (four in total) are conducted based on the data from eight countries, two experiments are conducted to test the classification performance and the generalization. For the first type, data from all eight countries are mixed and randomly divided into a training set and a testing set with 75% data and 25% data, respectively. The ratio between training and testing set normally will have little effect on the classification performance, if both of them have adequate samples for each class. This data-splitting method is also applied for each country to test the performance of a specific country. For the second one, two 8-fold cross-validation experiments are further carried out. The first 8-fold cross-validation experiment is country-based, meaning seven countries are used as training data, and the remaining one is used as testing data in each fold, which is also known as Leave-One-Out Cross-Validation (LOOCV). For a deep discussion of the influence from the distribution of the dataset, the second 8-fold cross-validation is distribution-based, which means that all data are mixed and divided into eight folds and each fold contains data from all eight countries according to the ratio they have in all samples.

Overall accuracy (ACC) and the area under the receiver operating characteristic curve (AUC) are used to validate the overall performance, while precision (PR), recall (RE), and F1-score (F1) are used to evaluate the performance for each class. Python is the primary language for conducting experiments on the Alienware R13, equipped with an Intel i9 processor, 64GB of RAM, dual 2 TB SSDs, and an RTX 3090 graphics card. Code and processed data can be accessed through GitHub,^3^ once our paper is published.

## 3 Results

### 3.1 Experiment 1

Table 2 shows the classification performance compared among four models on HIV and STI identification tasks and each experimental result is from the setting that all data is mixed and divided into a training set and a testing set with 75% data and 25% data, respectively. For both HIV and STI classification tasks without resampling methods, performances are quite low for the positive samples, who have HIV or STI. However, the classification performances are improved when resampling methods, especially the SMOTE algorithm, are applied. One reason to explain this phenomenon is that the ratio of positive samples is too low to be identified, which also leads to the ‘*high*’ performance of negative samples. This invalid classification effectiveness can be easily found from the low AUC and positive results without the resampling method in Table 2.

**Table 2:** Classification performance comparison among four models on HIV and STI task.

Task	Resample method	Model	ACC	AUC	PR		RE		F1	
					Negative	Positive	Negative	Positive	Negative	Positive
HIV	No resample	RF	0.9904	0.6939	0.9909	0	0.9995	0	0.9952	0
		GBM	0.9904	0.6396	0.9909	0	0.9995	0	0.9952	0
		XGBoost	0.9907	0.6779	0.9909	0	0.9998	0	0.9953	0
		GBM-RF-Xgboost	0.9904	0.4995	0.9909	0	0.9995	0	0.9952	0
	SMOTE	RF	0.9849	0.9971	0.9946	0.9757	0.9751	0.9947	0.9848	0.9851
		GBM	0.8755	0.9429	0.9059	0.8495	0.8375	0.9134	0.8704	0.8803
		XGBoost	0.9105	0.9670	0.9435	0.8823	0.8729	0.9479	0.9068	0.9139
		GBM-RF-Xgboost	0.9849	0.9872	0.9946	0.9757	0.9751	0.9947	0.9848	0.9851
	Over-sampling + under-sampling	RF	0.9359	0.9855	0.9572	0.9138	0.9204	0.9535	0.9384	0.9332
		GBM	0.8619	0.9352	0.8938	0.8302	0.8394	0.8873	0.8657	0.8578
		XGBoost	0.8912	0.9569	0.9270	0.8564	0.8630	0.9232	0.8938	0.8885
		GBM-RF-Xgboost	0.9359	0.9507	0.9572	0.9138	0.9204	0.9535	0.9384	0.9332
STI	No resample	RF	0.9256	0.5693	0.9308	0.1190	0.9939	0.0110	0.9613	0.0201
		GBM	0.9301	0.6005	0.9307	0.2778	0.9993	0.0037	0.9638	0.0072
		XGBoost	0.9298	0.5897	0.9307	0.2308	0.9989	0.0044	0.9636	0.0086
		GBM-RF-Xgboost	0.9256	0.5043	0.9308	0.1190	0.9939	0.0110	0.9613	0.0201
	SMOTE	RF	0.8964	0.9557	0.9196	0.8757	0.8685	0.9242	0.8933	0.8993
		GBM	0.7189	0.7975	0.7230	0.7150	0.7088	0.7290	0.7159	0.7219
		XGBoost	0.7404	0.8232	0.7480	0.7333	0.7243	0.7565	0.7360	0.7447
		GBM-RF-Xgboost	0.8964	0.9058	0.9196	0.8757	0.8685	0.9242	0.8933	0.8993
	Over-sampling + under-sampling	RF	0.7380	0.8133	0.7558	0.7192	0.7413	0.7345	0.7484	0.7267
		GBM	0.6614	0.7229	0.6645	0.6573	0.7189	0.5976	0.6906	0.6260
		XGBoost	0.6717	0.7296	0.6795	0.6622	0.7108	0.6284	0.6948	0.6448
		GBM-RF-Xgboost	0.7380	0.7628	0.7558	0.7192	0.7413	0.7345	0.7484	0.7267

^*^ACC, accuracy; PR, Precision; RE, recall; F1, F1-score.

Due to the fact that the positive ratio is extremely low, there is a very high probability that the new data generated through resampling methods, especially the SMOTE method, will be clustered in an extremely small area, which means that there is a very high probability that there is a high overlap of the positive samples between the training set and the testing set. An obvious factor to be evaluated is the resampling strategy, i.e., the ratio of the number of samples in the minority class to the number of samples in the majority class after resampling. Based on the experiment above, an expanded experiment is conducted, whose resampling strategy is changed from 0.2 to 1. Table 3 shows the comparison among four models on HIV and STI tasks with different resampling strategies. It can be easily summarized that the resampling strategy has some positive contribution to the classification performance improvement, but not so much as expected, especially for the RF and GBM-RF-Xgboost model, which also means that the improvement is not from the possible overlap between the training set and testing set, which is created by resampling.

**Table 3:** Classification performance comparison among four models on HIV and STI task with different resampling strategy.

Task	Resampling strategy	Model	ACC	AUC	PR		RE		F1	
					Negative	Positive	Negative	Positive	Negative	Positive
HIV	0.2	RF	0.9744	0.9910	0.9861	0.9148	0.9834	0.9277	0.9847	0.9212
		GBM	0.9045	0.9363	0.9201	0.7847	0.9704	0.5612	0.9446	0.6544
		XGBoost	0.9269	0.9620	0.9438	0.8208	0.9707	0.6993	0.9571	0.7552
		GBM-RF-Xgboost	0.9744	0.9672	0.9861	0.9148	0.9834	0.9277	0.9847	0.9212
	0.5	RF	0.9799	0.9962	0.9918	0.9570	0.9780	0.9837	0.9849	0.9702
		GBM	0.8745	0.9426	0.9035	0.8150	0.9092	0.8047	0.9064	0.8098
		XGBoost	0.9080	0.9667	0.9372	0.8516	0.9242	0.8754	0.9306	0.8633
		GBM-RF-Xgboost	0.9799	0.9851	0.9918	0.9570	0.9780	0.9837	0.9849	0.9702
	0.7	RF	0.9806	0.9967	0.9912	0.9663	0.9752	0.9880	0.9832	0.9770
		GBM	0.8714	0.9431	0.8996	0.8344	0.8767	0.8640	0.8880	0.8490
		XGBoost	0.9076	0.9670	0.9364	0.8709	0.9025	0.9148	0.9191	0.8923
		GBM-RF-Xgboost	0.9806	0.9849	0.9912	0.9663	0.9752	0.9880	0.9832	0.9770
	1.0	RF	0.9849	0.9971	0.9946	0.9757	0.9751	0.9947	0.9848	0.9851
		GBM	0.8755	0.9429	0.9059	0.8495	0.8375	0.9134	0.8704	0.8803
		XGBoost	0.9105	0.9670	0.9435	0.8823	0.8729	0.9479	0.9068	0.9139
		GBM-RF-Xgboost	0.9849	0.9872	0.9946	0.9757	0.9751	0.9947	0.9848	0.9851
STI	0.2	RF	0.8783	0.8220	0.8966	0.7110	0.9660	0.4291	0.9300	0.5352
		GBM	0.8424	0.7226	0.8464	0.6416	0.9915	0.0783	0.9132	0.1396
		XGBoost	0.8450	0.7453	0.8511	0.6422	0.9875	0.1145	0.9142	0.1944
		GBM-RF-Xgboost	0.8783	0.7099	0.8966	0.7110	0.9660	0.4291	0.9300	0.5352
	0.5	RF	0.8775	0.9225	0.9032	0.8235	0.9149	0.8019	0.9090	0.8126
		GBM	0.7454	0.7863	0.7609	0.6856	0.9031	0.4269	0.8259	0.5262
		XGBoost	0.7607	0.8108	0.7773	0.7037	0.9001	0.4790	0.8342	0.5700
		GBM-RF-Xgboost	0.8775	0.8728	0.9032	0.8235	0.9149	0.8019	0.9090	0.8126
	0.7	RF	0.8833	0.9407	0.9091	0.8488	0.8892	0.8749	0.8991	0.8616
		GBM	0.7234	0.7919	0.7354	0.7008	0.8231	0.5833	0.7768	0.6367
		XGBoost	0.7416	0.8168	0.7567	0.7151	0.8222	0.6280	0.7881	0.6688
		GBM-RF-Xgboost	0.8833	0.8958	0.9091	0.8488	0.8892	0.8749	0.8991	0.8616
	1.0	RF	0.8964	0.9557	0.9196	0.8757	0.8685	0.9242	0.8933	0.8993
		GBM	0.7189	0.7975	0.7230	0.7150	0.7088	0.7290	0.7159	0.7219
		XGBoost	0.7404	0.8232	0.7480	0.7333	0.7243	0.7565	0.7360	0.7447
		GBM-RF-Xgboost	0.8964	0.9058	0.9196	0.8757	0.8685	0.9242	0.8933	0.8993

^*^ACC, accuracy; PR, Precision; RE, recall; F1, F1-score.

**Figure 4:**
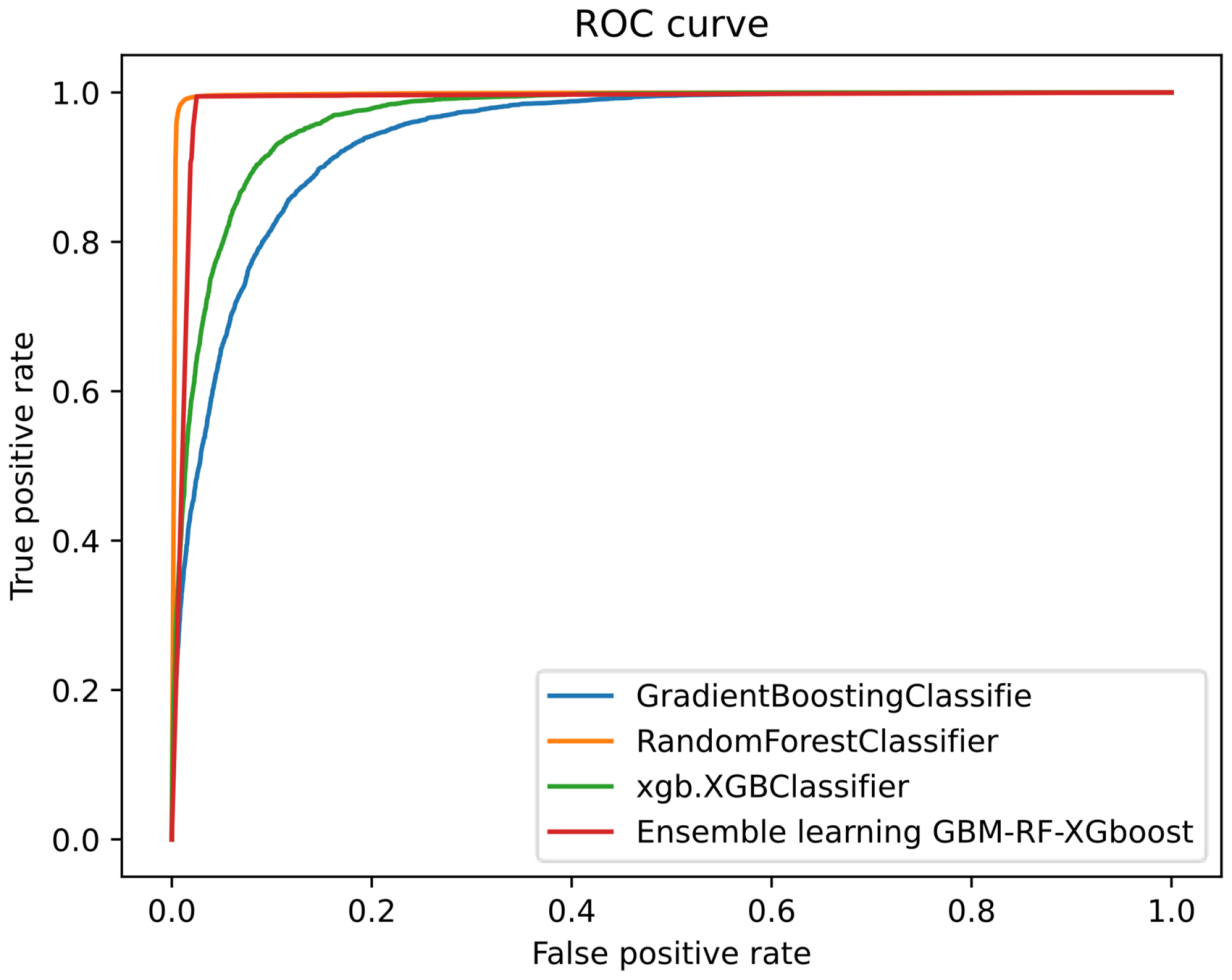
An example of ROC curve (resampling strategy = 1).

Table 2 and Figure 4 also demonstrate another interesting phenomenon, namely, all classification performances of the RF and the GBM-RF-Xgboost model are the same, except for the AUC. One explanation is that this ensemble model determines the prediction results from all three base learners, which means that it has more potential to output the prediction results, which is close to the model with the highest performance. This phenomenon is also observed in other following experiments.

### 3.2 Experiment 2

To further evaluate the classification performance on a single country data, experiments are also conducted based on each selected country as well. For each country, data is randomly split into a training set and a testing set according to 75 and 25%, respectively. Considering the effectiveness of the SMOTE method proved above, all results presented are those with the SMOTE resampling method. Table 4 demonstrates the classification results on HIV and STI, respectively. It can be easily observed that the different models can predict positive patients with similar performance levels. In terms of accuracy, we achieve a level of 0.9713 for HIV prediction and 0.8924 for STI prediction, respectively, when averaging across all the models and all the countries. The performance of these single-country-based experiments is close to but higher than the all-country-based experiments, whose average accuracy is 0.9390 for HIV classification and 0.8130 for STI classification, respectively. A possible explanation for this difference in performance is that the distribution of data, or culture, education, economy, etc., to be more specific, are quite far away from each other. As a result, classification abilities are enhanced when data from the same distribution but decreased when data from a different distribution. To evaluate the effects of the distribution factor, two 8-fold cross-validation experiments are conducted on these selected eight countries.

**Table 4:** Detail HIV and STI risk prediction for each country.

Task	Country	Model	ACC	AUC	PR		RE		F1	
					Negative	Positive	Negative	Positive	Negative	Positive
HIV	Angola	RF	0.9784	0.9982	0.9895	0.9673	0.9679	0.9893	0.9786	0.9782
		GBM	0.9659	0.9948	0.9932	0.9405	0.9396	0.9933	0.9657	0.9662
		XGBoost	0.9764	0.9960	0.9960	0.9576	0.9576	0.9960	0.9764	0.9764
		GBM-RF-Xgboost	0.9823	0.9867	0.9896	0.9750	0.9756	0.9893	0.9825	0.9821
	Dominican Republic	RF	0.9645	0.9949	0.9808	0.9479	0.9503	0.9798	0.9653	0.9636
		GBM	0.9661	0.9934	0.9839	0.9481	0.9503	0.9832	0.9668	0.9653
		XGBoost	0.9661	0.9956	0.9808	0.9510	0.9534	0.9798	0.9669	0.9652
		GBM-RF-Xgboost	0.9693	0.9816	0.9779	0.9603	0.9627	0.9764	0.9703	0.9683
	Dominican	RF	0.9840	0.9981	0.9918	0.9765	0.9763	0.9918	0.9840	0.9841
		GBM	0.9687	0.9945	0.9902	0.9488	0.9470	0.9906	0.9682	0.9692
		XGBoost	0.9781	0.9967	0.9917	0.9651	0.9645	0.9918	0.9779	0.9783
		GBM-RF-Xgboost	0.9840	0.9897	0.9918	0.9765	0.9763	0.9918	0.9840	0.9841
	Ethiopia	RF	0.9841	0.9982	0.9950	0.9737	0.9734	0.9950	0.9840	0.9842
		GBM	0.9725	0.9941	0.9898	0.9562	0.9551	0.9901	0.9722	0.9729
		XGBoost	0.9810	0.9951	0.9964	0.9663	0.9656	0.9965	0.9808	0.9812
		GBM-RF-Xgboost	0.9841	0.9882	0.9950	0.9737	0.9734	0.9950	0.9840	0.9842
	Guinea	RF	0.9880	0.9994	0.9930	0.9828	0.9838	0.9925	0.9884	0.9876
		GBM	0.9832	0.9964	0.9906	0.9755	0.9769	0.9900	0.9837	0.9827
		XGBoost	0.9856	0.9974	0.9953	0.9756	0.9769	0.9950	0.9860	0.9852
		GBM-RF-Xgboost	0.9856	0.9928	0.9930	0.9779	0.9792	0.9925	0.9860	0.9852
	Haiti	RF	0.9671	0.9939	0.9838	0.9514	0.9501	0.9843	0.9667	0.9675
		GBM	0.8996	0.9653	0.9271	0.8752	0.8683	0.9312	0.8967	0.9023
		XGBoost	0.9397	0.9830	0.9692	0.9134	0.9087	0.9709	0.9380	0.9413
		GBM-RF-Xgboost	0.9671	0.9740	0.9838	0.9514	0.9501	0.9843	0.9667	0.9675
	India	RF	0.9919	0.9985	0.9970	0.9868	0.9869	0.9970	0.9920	0.9919
		GBM	0.9501	0.9883	0.9711	0.9305	0.9287	0.9718	0.9494	0.9507
		XGBoost	0.9753	0.9958	0.9915	0.9597	0.9592	0.9917	0.9751	0.9754
		GBM-RF-Xgboost	0.9919	0.9936	0.9970	0.9868	0.9869	0.9970	0.9920	0.9919
	Cameroon	RF	0.9674	0.9951	0.9912	0.9453	0.9442	0.9913	0.9671	0.9678
		GBM	0.9546	0.9868	0.9811	0.9303	0.9284	0.9816	0.9540	0.9552
		XGBoost	0.9605	0.9917	0.9834	0.9390	0.9379	0.9838	0.9601	0.9609
		GBM-RF-Xgboost	0.9674	0.9813	0.9912	0.9453	0.9442	0.9913	0.9671	0.9678
STI	Angola	RF	0.9007	0.9657	0.9235	0.8797	0.8759	0.9260	0.8991	0.9023
		GBM	0.8746	0.9446	0.9028	0.8495	0.8424	0.9075	0.8716	0.8776
		XGBoost	0.8859	0.9554	0.9063	0.8670	0.8633	0.9090	0.8843	0.8875
		GBM-RF-Xgboost	0.9007	0.9292	0.9235	0.8797	0.8759	0.9260	0.8991	0.9023
	Dominican Republic	RF	0.9643	0.9939	0.9843	0.9428	0.9486	0.9825	0.9662	0.9622
		GBM	0.9513	0.9920	0.9839	0.9180	0.9245	0.9825	0.9533	0.9492
		XGBoost	0.9594	0.9923	0.9811	0.9362	0.9426	0.9789	0.9615	0.9571
		GBM-RF-Xgboost	0.9675	0.9801	0.9814	0.9522	0.9577	0.9789	0.9694	0.9654
	Dominican	RF	0.9673	0.9946	0.9809	0.9547	0.9526	0.9817	0.9665	0.9680
		GBM	0.9194	0.9750	0.9504	0.8929	0.8835	0.9547	0.9157	0.9227
		XGBoost	0.9448	0.9872	0.9677	0.9244	0.9193	0.9698	0.9429	0.9466
		GBM-RF-Xgboost	0.9673	0.9772	0.9809	0.9547	0.9526	0.9817	0.9665	0.9680
	Ethiopia	RF	0.9675	0.9952	0.9838	0.9520	0.9510	0.9842	0.9671	0.9678
		GBM	0.9318	0.9768	0.9579	0.9082	0.9041	0.9597	0.9302	0.9332
		XGBoost	0.9536	0.9899	0.9733	0.9351	0.9332	0.9741	0.9529	0.9542
		GBM-RF-Xgboost	0.9675	0.9813	0.9838	0.9520	0.9510	0.9842	0.9671	0.9678
	Guinea	RF	0.8758	0.9386	0.8854	0.8655	0.8763	0.8754	0.8808	0.8704
		GBM	0.8502	0.9354	0.8616	0.8380	0.8505	0.8499	0.8560	0.8439
		XGBoost	0.8610	0.9395	0.8800	0.8415	0.8505	0.8725	0.8650	0.8567
		GBM-RF-Xgboost	0.8758	0.9122	0.8854	0.8655	0.8763	0.8754	0.8808	0.8704
	Haiti	RF	0.8900	0.9542	0.9070	0.8736	0.8743	0.9065	0.8903	0.8897
		GBM	0.7733	0.8590	0.7835	0.7630	0.7680	0.7787	0.7757	0.7708
		XGBoost	0.8146	0.8963	0.8333	0.7968	0.7961	0.8340	0.8143	0.8150
		GBM-RF-Xgboost	0.8900	0.9069	0.9070	0.8736	0.8743	0.9065	0.8903	0.8897
	India	RF	0.8912	0.9529	0.9184	0.8669	0.8609	0.9221	0.8887	0.8936
		GBM	0.7271	0.8013	0.7338	0.7205	0.7203	0.7340	0.7270	0.7272
		XGBoost	0.7513	0.8327	0.7612	0.7419	0.7389	0.7640	0.7499	0.7528
		GBM-RF-Xgboost	0.8912	0.9056	0.9184	0.8669	0.8609	0.9221	0.8887	0.8936
	Cameroon	RF	0.8714	0.9477	0.8774	0.8658	0.8598	0.8828	0.8685	0.8742
		GBM	0.8371	0.9089	0.8407	0.8337	0.8269	0.8471	0.8337	0.8404
		XGBoost	0.8610	0.9261	0.8640	0.8581	0.8528	0.8690	0.8583	0.8635
		GBM-RF-Xgboost	0.8714	0.9028	0.8774	0.8658	0.8598	0.8828	0.8685	0.8685

^*^ACC, accuracy; PR, Precision; RE, recall; F1, F1-score.

### 3.3 Experiment 3

The first k-fold cross-validation is country-based LOOCV (Leave-one-out cross-validation). For each fold, data is resampled using the SMOTE method first, and then seven countries are selected to construct a training set, while the remaining one is used as a validation set. The process will repeat until all eight countries are tested. Table 5 shows the country-based LOOCV evaluation results for HIV samples. The performance of all models decreases, with the lowest accuracy being 0.5356. This unacceptable result is closer to randomly selecting one of two labels, rather than predicting results by learning features. The reason for the performance reduction is that the distribution of the validation set is totally different from the training set for each fold. This phenomenon is also observed in the country-based LOOCV for STI classification.

**Table 5:** Country-based 8-fold leave-one-out cross-validation (HIV).

Fold	Model	ACC	AUC	PR		RE		F1	
				Negative	Positive	Negative	Positive	Negative	Positive
#1	RF	0.6601	0.7797	0.6032	0.8568	0.9358	0.3844	0.7335	0.5307
	GBM	0.7587	0.8506	0.7875	0.7350	0.7084	0.8089	0.7459	0.7702
	XGBoost	0.7492	0.8338	0.7338	0.7668	0.7821	0.7163	0.7572	0.7407
	GBM-RF-Xgboost	0.6601	0.7979	0.6032	0.8568	0.9358	0.3844	0.7335	0.5307
#2	RF	0.5356	0.6640	0.5186	0.8986	0.9909	0.0802	0.6809	0.1473
	GBM	0.6426	0.7786	0.5882	0.8718	0.9508	0.3344	0.7268	0.4834
	XGBoost	0.6488	0.7293	0.5927	0.8758	0.9508	0.3467	0.7303	0.4968
	GBM-RF-Xgboost	0.5356	0.6631	0.5186	0.8986	0.9909	0.0802	0.6809	0.1473
#3	RF	0.5982	0.8212	0.5584	0.8105	0.9401	0.2564	0.7006	0.3896
	GBM	0.7591	0.8468	0.7902	0.7340	0.7055	0.8126	0.7454	0.7713
	XGBoost	0.7464	0.8173	0.7260	0.7709	0.7916	0.7013	0.7574	0.7344
	GBM-RF-Xgboost	0.5982	0.7864	0.5584	0.8105	0.9401	0.2564	0.7006	0.3896
#4	RF	0.5342	0.7141	0.5179	0.8680	0.9877	0.0806	0.6795	0.1475
	GBM	0.6529	0.7748	0.5995	0.8302	0.9214	0.3845	0.7264	0.5256
	XGBoost	0.6157	0.7566	0.5700	0.8332	0.9421	0.2894	0.7103	0.4295
	GBM-RF-Xgboost	0.5342	0.6601	0.5179	0.8680	0.9877	0.0806	0.6795	0.1475
#5	RF	0.6236	0.8241	0.5721	0.9313	0.9803	0.2668	0.7225	0.4148
	GBM	0.8472	0.9026	0.8326	0.8630	0.8690	0.8253	0.8504	0.8438
	XGBoost	0.8033	0.8924	0.7508	0.8835	0.9079	0.6987	0.8219	0.7803
	GBM-RF-Xgboost	0.6236	0.8617	0.5721	0.9313	0.9803	0.2668	0.7225	0.4148
#6	RF	0.5468	0.7358	0.5251	0.8376	0.9775	0.1160	0.6832	0.2038
	GBM	0.6884	0.7771	0.6370	0.8014	0.8758	0.5010	0.7376	0.6165
	XGBoost	0.6415	0.7448	0.5935	0.7913	0.8986	0.3845	0.7148	0.5175
	GBM-RF-Xgboost	0.5468	0.6916	0.5251	0.8376	0.9775	0.1160	0.6832	0.2038
#7	RF	0.5518	0.7518	0.5281	0.8301	0.9734	0.1302	0.6847	0.2250
	GBM	0.6845	0.7926	0.6266	0.8394	0.9127	0.4562	0.7431	0.5911
	XGBoost	0.6486	0.7497	0.5955	0.8349	0.8267	0.3706	0.7251	0.5133
	GBM-RF-Xgboost	0.5518	0.6899	0.5281	0.8301	0.9734	0.1302	0.6847	0.2250
#8	RF	0.5603	0.7417	0.5331	0.8441	0.9727	0.1480	0.6887	0.2518
	GBM	0.7154	0.8142	0.6724	0.7871	0.8403	0.5905	0.7470	0.6748
	XGBoost	0.6890	0.7965	0.6363	0.8084	0.8826	0.4955	0.7395	0.6144
	GBM-RF-Xgboost	0.5603	0.7290	0.5331	0.8441	0.9727	0.1480	0.6887	0.2518

^*^ACC, accuracy; PR, Precision; RE, recall; F1, F1-score.

### 3.4 Experiment 4

To further evaluate the generalization of all four models and demonstrate the effect of distribution, another data-ratio-based 8-fold cross-validation is carried out on these selected eight countries. In this experiment, data is resampled with the SMOTE method first to solve the data imbalanced issue, after which, the dataset of each country is divided into 8 segments averagely, and coded with 1, 2, …, 8. For the *i*-th cross-validation, segments coded with *i* from all countries are selected and stacked together to construct a validation set, while the remaining segments are stacked to form a training set. This process will be repeated until all 8 segments of each country are tested. This data split method ensures that the distribution of the training set and the testing set are close or even the same and the ratio of data from a country keeps the same in the training set and the validation set. Table 6 shows the ratio-based classification performance, where we can find that the average accuracy of all eight folds achieves 0.9382, which is the same level as the result of experiment 1.

**Table 6:** Ratio-based 8-fold leave-one-out cross-validation (HIV).

Fold	Model	ACC	AUC	PR		RE		F1	
				Negative	Positive	Negative	Positive	Negative	Positive
#1	RF	0.9786	0.9963	0.9889	0.9683	0.9688	0.9887	0.9787	0.9784
	GBM	0.8812	0.9505	0.9029	0.8609	0.8592	0.9041	0.8805	0.8820
	XGBoost	0.9149	0.9713	0.9420	0.8899	0.8874	0.9434	0.9139	0.9158
	GBM-RF-Xgboost	0.9786	0.9836	0.9889	0.9683	0.9688	0.9887	0.9787	0.9784
#2	RF	0.9789	0.9961	0.9880	0.9702	0.9693	0.9883	0.9786	0.9792
	GBM	0.8776	0.9499	0.8944	0.8623	0.8548	0.9001	0.8742	0.8808
	XGBoost	0.9155	0.9712	0.9370	0.8962	0.8900	0.9408	0.9129	0.9180
	GBM-RF-Xgboost	0.9789	0.9828	0.9880	0.9702	0.9693	0.9883	0.9786	0.9792
#3	RF	0.9773	0.9955	0.9874	0.9676	0.9670	0.9876	0.9771	0.9775
	GBM	0.8789	0.9482	0.8965	0.8627	0.8570	0.9008	0.8763	0.8814
	XGBoost	0.9140	0.9701	0.9363	0.8938	0.8887	0.9394	0.9119	0.9161
	GBM-RF-Xgboost	0.9773	0.9816	0.9874	0.9676	0.9670	0.9876	0.9771	0.9775
#4	RF	0.9804	0.9965	0.9888	0.9723	0.9720	0.9889	0.9803	0.9805
	GBM	0.8792	0.9485	0.8951	0.8644	0.8599	0.8986	0.8772	0.8811
	XGBoost	0.9132	0.9719	0.9328	0.8951	0.8911	0.9354	0.9115	0.9148
	GBM-RF-Xgboost	0.9804	0.9842	0.9888	0.9723	0.9720	0.9889	0.9803	0.9805
#5	RF	0.9800	0.9961	0.9875	0.9727	0.9721	0.9877	0.9797	0.9802
	GBM	0.8763	0.9474	0.8913	0.8625	0.8560	0.8964	0.8733	0.8791
	XGBoost	0.9144	0.9704	0.9359	0.8951	0.8891	0.9395	0.9119	0.9168
	GBM-RF-Xgboost	0.9800	0.9829	0.9875	0.9727	0.9721	0.9877	0.9797	0.9802
#6	RF	0.9798	0.9962	0.9862	0.9736	0.9731	0.9864	0.9796	0.9800
	GBM	0.8792	0.9510	0.8946	0.8650	0.8590	0.8992	0.8764	0.8818
	XGBoost	0.9166	0.9730	0.9374	0.8979	0.8925	0.9407	0.9144	0.9188
	GBM-RF-Xgboost	0.9798	0.9845	0.9862	0.9736	0.9731	0.9864	0.9796	0.9800
#7	RF	0.9800	0.9969	0.9879	0.9725	0.9715	0.9883	0.9796	0.9804
	GBM	0.8811	0.9503	0.8993	0.8649	0.8555	0.9062	0.8769	0.8850
	XGBoost	0.9150	0.9708	0.9396	0.8935	0.8852	0.9442	0.9116	0.9182
	GBM-RF-Xgboost	0.9800	0.9839	0.9879	0.9725	0.9715	0.9883	0.9796	0.9804
#8	RF	0.9788	0.9956	0.9861	0.9719	0.9713	0.9863	0.9786	0.9790
	GBM	0.8812	0.9506	0.9003	0.8639	0.8568	0.9055	0.8780	0.8842
	XGBoost	0.9163	0.9707	0.9370	0.8975	0.8922	0.9402	0.9141	0.9184
	GBM-RF-Xgboost	0.9788	0.9829	0.9861	0.9719	0.9713	0.9863	0.9786	0.9790

^*^ACC, accuracy; PR, Precision; RE, recall; F1, F1-score.

## 4 Discussion

From experiment 1, experiment 2, experiment 3 and experiment 4, we can draw some simple but important conclusions:

1. Data imbalance problem is a very crucial factor in STIs/HIV identification tasks, while data resampling techniques may help to improve the classification performance of all models. However, compared with the combination of over-sampling the minor class and under-sampling the primary class, the SMOTE method has more advantages in enhancing performance. The increased ratio of positive data points enables classifiers to have more chances to learn the features of positive samples and classify them correctly, which leads to the improvement of the classification performance. An inevitable factor contributing to machine learning algorithms is the size of the dataset. Therefore, even though the combined resampling method increases the positive ratio, its reduction of dataset size by under-sampling the negative samples limit its application in the STIs/HIV identification task. This also explains why the SMOTE outperforms the combined method.
2. The resampling technique generates new data points by calculating the relationships among existing data points. Therefore, there is little space to generate new data if the minor class accounts for an extremely small percentage, which means that there is a higher probability that the coordinates of two data points generated in different steps may be too close or even the same, which leads to a higher overlap between training sets and testing sets, and finally limits the generalization of models and performance. However, Table 3 demonstrates that the performance improves sharply when SMOTE is applied, though only a little data is generated. The performance improvement speed rate decreases with more generated data, which is evidence that a higher overlap rate has little effect on performance enhancement.
3. RF and the ensemble GBM-RF-Xgboost model outperform all other algorithms in most experiments, except the country-based k-fold cross-validation, which indicates that both the RF and the ensemble model can be easily influenced by the distribution of data.
4. The distribution of data plays an important role in the STIs/HIV classification task, which represents the different cultures, education levels, economy, attitude of sex, etc. These factors may vary from country to country. The comparison between experiment 1, experiment 3, and experiment 4 demonstrates the simple truth that all models perform much better if data is from the same distribution, while totally different distributions between training sets and testing sets may heavily decrease the classification performance and the reduction of identification abilities cannot be enhanced by changing the dataset size or ratio among all categories.

**Figure 5:**
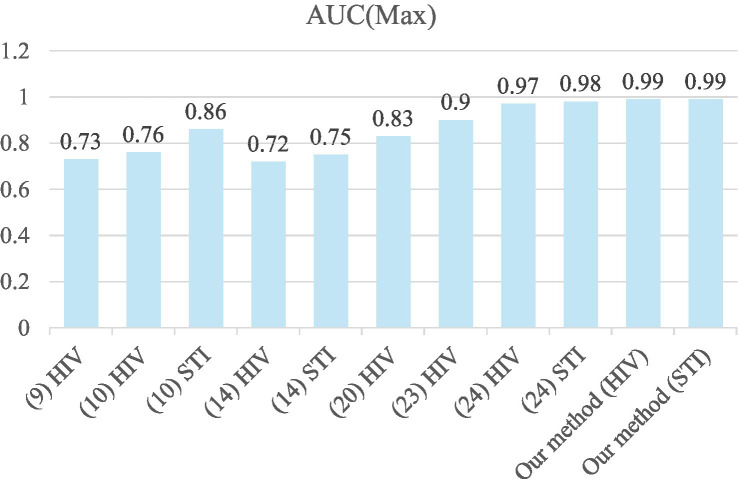
Comparison of max AUC among recent studies on HIV/STI prediction.

Figure 5 shows the comparison of max AUC among recent studies on STIs/HIV prediction. Our results, with an AUC reaching up to 0.99 for both HIV (Angola, RF) and STI (Ethiopia, RF), are considered excellent outcomes in the assessment of diagnostic tests in medical applications, as reported by Mandrekar (30). Our prediction results also outperform recently reported works (9, 10, 14, 20, 23, 24) for STI/HIV risk predictions. This may be due to our proposed five-step system for analyzing the data to remove missing, overlapped, and unimportant features. A five-step data analysis process is conducted for data preparation before feeding them to machine learning models (GBM, RF, XG Boost, Ensemble learning) to reduce the computation time for the model training. The five-step system also consists of the imbalanced data-solving part. When we applied SMOTE to the experimental data, the highest prediction performance was considered outstanding, with an AUC above 0.99 and an accuracy of 99%. The over-sampled method is applied to the minor class based on its distribution in the SMOTE technique. This means more data samples of the minor class were created and the majority class was kept as its original data. Before building the model, data imbalance was solved effectively to train models. Furthermore, our data analysis method can be applied to similar problems that use Electronic Health Records (EHR) data to predict the outcome of any health conditions.

The main intention of this study was to build a model from public data that can be used for any individual independent clinic or service. The results of our experiment demonstrate excellent potential for building a model from multi-country data. The model can be used for private stakeholders who are quite often vulnerable. The predictive model is not meant to be used to replace clinical judgment by clinical health experts in managing and treating STIs/HIV. Instead, it can be used as an automated, thereby more efficient, screening tool to identify those people at high risk for primary or secondary infection with STIs/HIV. It may also help to overcome biases impacting judgment among clinicians or overcoming elements of human error etc. It also has implications for efficiencies in health service delivery. The predictive results can work as a prompt in the decision-making process to consider further screening and testing for those in high-risk groups, as well as for initiating further actions related to health promotion, sexual health education, harm reduction, and linkage to supports and services. The STIs/HIV risk prediction model could also have relevance for underserved groups who may be less likely to access mainstream health services and who may be at greater risk for STIs/HIV; and lend itself well to alternate modes of delivery of care to harder-to-reach groups (E.G., point of care testing, mobile outreach, via peer-workers, self-testing).

This research is a demonstration study of gathering a large amount of data from multiple countries to build a more generalized predictive model. Moreover, our five-step data pre-processing plan can make training the model for STIs/HIV prediction more efficient.

There are some limitations in the proposed approach, which are mainly related to the datasets used to build the model. (1) Although the DHS data source is comprehensive and includes data from 90 countries, the data used in this study was limited to the selected Asian and African countries because public data was not available from the other countries on STIs/HIV status or related behaviors. This may have implications of lower accuracy for STIs/HIV prediction in Western countries or higher-income countries. Future research can refine the model and may see further improvements to the global model if and when more data becomes available from all continents. (2) The data collection consistency in time also limits its application. In this study, only data collected in recent years is discussed. However, human behaviors may change heavily as time passes, which means that a current acceptable model in a specific place may be dysfunctional in the future or in a different place. A more flexible and robust model considering time factor is worth exploring. (3) Underreporting in self-assessments or self-reported surveys on sensitive and personal topics such as STIs/HIV status is common (31). A US study of 165,828 outpatient visits (32), found that people tended to conceal their interest in HIV testing when presenting to the clinic, despite that this may have been their primary reason for attending. Since most data features or attributes from the primary data sets are obtained from self-reported information provided by clients. (4) This study only considered variables which are classic and well-recognized in STI research, while some variables that are currently relevant, particularly those sensitive to specific policies aimed at reducing STI infections in populations such as men who have sex with men, were not included in this study.

In the initial stages, concealing or under-reporting stigmatized, high-risk behaviors due to fear of stigma or discrimination regarding STIs/HIV testing, diagnosis, and treatment-seeking could introduce data uncertainty issues, resulting in lower accuracy of a model or less reliable analysis. Third, the datasets used in this study only consisted of data from men, as that was our target population. Therefore, the trained model reported in this paper may not work for other population groups, such as adolescents and women, in terms of STIs/HIV risk prediction. Other more nuanced behaviors of subgroup populations, if not included in the model, may also have limited applicability, such as the impact of alcohol and other substance use or abuse on STIs/HIV risk or the role of social networks in STIs/HIV transmission.

Despite the limitation of not considering specific risk factors and behaviors unique to the population of men who have sex with men, our framework for building a suitable predictive model for any population remains reliable and dependable. It allows for a systematic and step-by-step approach to develop and validate a universal model that can accurately predict STIs/HIV risk. Future studies could include data from other population groups, such as adolescents or women, to further improve the model’s accuracy and applicability to a wider range of individuals.

## 5 Conclusion

HIV and STIs continue to pose significant global public health challenges. While emerging machine learning methods have shown effectiveness in predicting the risk of STIs/HIV compared to traditional multivariable logistic regression-based approaches, they also come with their own set of challenges. A universal prediction model can streamline individual client risk prediction in local clinics and health services by eliminating the need to collect and store large datasets at the local level. We propose a universal prediction model and demonstrate the development and validation of a highly accurate STIs/HIV prediction model using publicly available data. This proposed model cannot only be integrated as a useful and efficient tool for enhancing the performance of predicting the risk of having an STI/HIV for affected individuals but also provides an effective strategy to identify individuals at high risk for HIV in different environments. Observed performance also indicates the need for building digital platforms to identify STI/HIV patients, which leads to the effective reallocation of medical resources and cost savings from management in policy.

## Data Availability

Publicly available datasets were analyzed in this study. This data can be found at: https://dhsprogram.com/.

## Footnotes

^1^ https://dhsprogram.com/

^2^ http://scikit-learn.org/stable/

^3^ https://github.com/xiaopengusq/STI-HIV-Risk-Prediction-Model
